# Metabolites of the gut microbiota may serve as precise diagnostic markers for sarcopenia in the elderly

**DOI:** 10.3389/fmicb.2023.1301805

**Published:** 2023-12-21

**Authors:** Yangli He, Weipeng Cui, Tuanyu Fang, Zeng Zhang, Min Zeng

**Affiliations:** ^1^Center of Geriatrics, Hainan General Hospital/Hainan Affiliated Hospital of Hainan Medical University, Haikou, Hainan Province, China; ^2^School of Food Science and Engineering, Key Laboratory of Food Nutrition and Functional Food of Hainan Province, Hainan University, Haikou, Hainan, China; ^3^Department of Endocrine, Hainan General Hospital/Hainan Affiliated Hospital of Hainan Medical University, Haikou, Hainan Province, China

**Keywords:** sarcopenia, gut metagenome, metabolomic, gut-muscle axis, gut microbiota

## Abstract

Sarcopenia, a disease recognized by the World Health Organization, has posed a great challenge to the world in the current aging society. The vital role of the gut microbiome through the gut-muscle axis in sarcopenia is increasingly recognized. However, the working mechanisms by which the gut microbiota functions have not been fully explored in the multi-omics field. Here, we designed a cross-sectional study that recruited patients (*n* = 32) with sarcopenia and healthy old adults (*n* = 31). Diagnosis of sarcopenia was based on the Asian Working Group for Sarcopenia (AWGS) in 2019 criteria. Muscle mass was represented by appendicular skeletal muscle mass measured by using direct segmental multi-frequency bioelectrical impedance and muscle strength was evaluated using the handgrip strength. The Short Physical Performance Battery, the 5-time Chair Stand Test, and the 4-metre Walk Test were used to assess physical performance. Shotgun metagenomic sequencing was used to profile the gut microbiome in order to identify its construction and function. Metabolome based on untargeted metabolomics was applied to describe the features and structure of fecal metabolites. In clinical indexes including triglycerides and high-density lipoprotein cholesterol, we noted a significant decrease in triglycerides (TG) and a significant increase in high-density lipoprotein cholesterol (HDL-C) in patients with sarcopenia. Appendicular skeletal muscle mass of patients with sarcopenia was lower than the health group. Based on intestinal metagenomic and fecal metabolomic profiles, we found that the gut microbiome and metabolome were disturbed in patients with sarcopenia, with significant decreases in bacteria such as *Bifidobacterium longum*, *Bifidobacterium pseudocatenulatum*, and *Bifidobacterium adolescentis*, as well as metabolites such as shikimic acid. Also, we plotted supervised classification models at the species level of gut bacteria (AUC = 70.83–88.33) and metabolites (AUC = 92.23–98.33) based on machine learning, respectively. Based on the gut-muscle axis network, a potential mechanism is proposed along the gut microbiome - key metabolites - clinical index, that *Phascolarctobacterium faecium* affects appendicular skeletal muscle mass, calf circumference, handgrip strength, and BMI via Shikimic acid metabolites. This study elucidates the potential mechanisms by which the gut microbiome influences the progress of sarcopenia through metabolites and provides a meaningful theoretical foundation for reference in the diagnosis and treatment of sarcopenia.

## 1 Introduction

Sarcopenia, defined as age-related progressive loss of skeletal muscle mass and function, was recognized as a disease by the World Health Organization (Anker et al., 2016). Sarcopenia has an incidence of 5–13% in the 60–70 years population, and this percentage continues to increase with age (Petermann-Rocha et al., 2022). Nowadays, the number of elderly people is increasing (Almohaisen et al., 2022), and sarcopenia will pose more serious challenges in the aging society. Some studies have shown that patients with sarcopenia have serious risks and lower quality of life, such as falls and fractures (Yeung et al., 2019). In this situation, the Writing Group for the European Working Group on Sarcopenia in Older People 2 (EWGSOP2) and the Asian Working Group on Sarcopenia (AWGS) modified the diagnosis of sarcopenia in 2019 (Cruz-Jentoft et al., 2019; Chen L. K. et al., 2020). The primary non-pharmacological method to increase muscle mass and function is resistance training (Burtscher et al., 2022), but older individuals frequently fail to comply with treatment regimens and are not physically suitable for their sustainability (Hawley-Hague et al., 2014). Increased protein consumption, antioxidant supplements, essential amino acids and their derivatives, and polyunsaturated fats appear to be advantageous to muscle mass and function in the elderly (Robinson et al., 2018). A recent study discovered a contrary result that the intake of high amounts of protein increases the risk of sarcopenia (Ni Lochlainn et al., 2023). However, information is scarce on the pathophysiologic processes that underpin sarcopenia, and no particular treatment plan, including pharmaceutical therapy, has been created (Cruz-Jentoft and Sayer, 2019). Thus, novel understandings of the mechanisms of muscle loss should be taken into account (Liu et al., 2021).

The human gut microbiota is composed of 10 to 100 trillion bacteria and plays an essential role in human health by participating in metabolic interactions and immunological responses of the host (Ursell et al., 2012). The human gut engages in a number of host functions and regulates intestinal contents at the interface with the external environment. The ecology of microbiota controls the organism’s nutritional status and can affect the process by which nutrients are absorbed through the bioconversion of dietary molecules (O’Connor, 2013). In fact, among its activities, the gut microbiota is involved in the production of micronutrients like vital vitamins and cofactors, the regulation of the immune system, the transformation of xenobiotics, the breakdown of complex lipids, proteins and polysaccharides into metabolite intermediates, the detoxification of waste products, and finally acts as a barrier against the spread of pathogens (Hooper et al., 2012; LeBlanc et al., 2013). Inflammatory milieu and host metabolism are two important biological processes that might be affected by abnormal microbiome compositional patterns (Clemente et al., 2018). Microbes may reduce food absorption, stimulate insulin resistance, and promote oxidative stress, all of which are negative to muscle mass and function, and through affecting intestinal permeability, energy metabolism, hormone production, systemic inflammation, and immunological response, gut microbiota appears to have a significant influence on the musculoskeletal system (Ticinesi et al., 2019). Gut microbiota dysbiosis, which implies a disturbance in the balance of microbial communities with an overrepresentation of opportunistic pathogens and a decrease in commensals and symbionts (Lustgarten, 2019), is particularly frequent in older individuals (Ticinesi et al., 2019). Conversely, those who age successfully, like centenarians, exhibit particular health markers in their intestinal microbiota, including an increased presence of bacteria with anti-inflammatory capabilities, such as *Bifidobacteria* (Arboleya et al., 2016; Biagi et al., 2017). The altered microbial composition may play a role in the onset of sarcopenia and aging muscles through the mediation of microorganisms of external chemical influences (Cabreiro et al., 2013; Picca et al., 2018). Micronutrients and metabolites generated from the gut microbiota can reach and act on muscle, and the ‘gut-muscle axis’ has been proposed to investigate this interaction (Ticinesi et al., 2017). However, these results were based on 16S rRNA sequencing, widely used to examine microbiota studies as a robust technic, but it has a limited capacity to distinguish between closely related species and is unable to provide precise data on the functioning capacities of the microbiome community (Fischbach and Segre, 2016). Shotgun metagenomic sequencing could provide species-level functional annotations of microbial communities (Franzosa et al., 2018). However, only a few researchers have used this technique to investigate the relationship between gut microbiota and sarcopenia at present. At the same time, non-clinical and single omics cannot provide a more accurate mapping in studies of gut microbiome affecting sarcopenia.

To address these challenges, we recruited 63 participants in this cross-sectional study and divided them into two groups: the sarcopenia group (SAR) and the healthy control group (CON) according to their clinical indexes (including those related to liver, kidney, and lipid metabolism in the blood samples) and muscle performance. The gut microbial features of SAR patients were described using shotgun metagenomic sequencing and inferred metabolomics. Metabolome based on untargeted metabolomics was applied to describe the features and structure of fecal metabolites in SAR. Then identified biomarkers, including differential bacteria (*n* = 20) and metabolites (*n* = 34) between SAR and CON, were utilized to construct the model to distinguish SAR and CON by using machine learning, respectively. Finally, we propose possible mechanisms that the gut microbiome influences the physical performance of SRA may via the route of “gut microbiome-fecal metabolome - host clinical indexes.”

## 2 Materials and methods

### 2.1 Research design and subject recruitment

This cross-sectional study cohort was composed of two groups with a total of 63 participants that were recruited from Haikou, China. Diagnosis of the sarcopenia was based on the Asian Working Group for Sarcopenia (AWGS) in 2019 criteria (Chen L. K. et al., 2020). Specifically, sarcopenia was diagnosed by meeting one of the following two conditions: low appendicular skeletal muscle mass (ASM) and low Physical performance, or low ASM and low muscle strength. Low muscle strength was classified as handgrip strength of less than 28 kg for males and 18 kg for females. The criteria for low physical performance include a 4-metre walk speed of fewer than 1.0 m/s, a score of 9 on the Short Physical Performance Battery (SPPB), or a time of more than 12 s on the 5-time chair stand test. It was defined as low muscle mass when the ASM value was less than 7 kg/m^2^ for men and less than 5.7 kg/m^2^ for women. All subjects were tested according to the above criteria and subsequently divided into control (CON group, *n* = 31) and sarcopenia groups (SAR group, *n* = 32) according to diagnostic criteria. The grip strength of participants’ dominant hand was tested with the dynamometer (CAMRY EH101, China). ASM measurement using bioelectrical impedance analysis (Body composition analyzer CP10A, Runcobo, China).

No participants used antibiotics within a month before collecting fecal samples. All participants fasted for 12 h before the collection of blood samples for the clinical examination of the Liver function tests (TP, ALB, PA, GLB, A/G, CRP), Cholesterol tests (TC, TG, HDL, LDL), Kidney function tests (SCR, UA, BUN), HGB and GLU. On the same day of clinical examination, fecal samples were collected from each participant. After determining the weight of the fecal samples, the sample protector (CW0592M, CWBIO, China) was applied at a one-part fecal sample to five-part sample protectors ratio, and the samples remained at −20°C until further steps. All 63 samples were used for both Whole-genome shotgun (WGS) sequencing and Metabolomics measurements.

### 2.2 DNA extraction and shotgun metagenomic sequencing

The DNA Fecal Mini Kit Produced by QIAamp® (Qiagen, Hilden, Germany) was employed to perform DNA extraction. The integrity of the DNA was evaluated through 0.8% agarose gel electrophoresis, while the spectrophotometric measurement was employed to determine the OD 260/280 ratio. All the DNA samples were used to shotgun metagenomic sequencing utilizing an Illumina HiSeq 2,500 device (Illumina, CA, United States) at Novogene Company (Beijing, China), employing stringent protocols to ensure accurate and comprehensive analysis. Libraries were generated with a targeted fragment length of approximately 300 bp. Paired-end reads were then generated, with 100 bp in both the forward and reverse directions. The reads underwent thorough quality control analysis using FastQC to ensure data quality. Subsequently, the reads were aligned to the human genome in order to eliminate any host DNA fragments.

### 2.3 Identification of microbial species, functional genes, and pathways

Species annotation of metagenome samples was performed using metaphan3 software (v3.0.14), and the relative abundance of each species among all samples was calculated. Meanwhile, we used HumanN3 software (v3.0.1) for qualitative and quantitative analysis of metabolic pathways in metagenome samples based on the UniRef90 database.

For CAZyme annotation, we first performed an overlapping assembly of the shotgun reads using MEGAHIT (v1.0) and default parameters. Subsequently, genes were predicted by Metagenemark software based on the assembled contigs. Based on the dbcan database, CAZyme genes were annotated out by default parameters. Meanwhile, the ensemble of genes was de-redundantly adopted by the cd-hit software. Based on bowtie software, we built library comparisons for the redundant post-existing genes and calculated the abundance of functional genes in each sample.

### 2.4 Untargeted metabolomics determination

The fecal sample, preserved in −80°C, was thawed in ice. A solution of 400 μL (Methanol: Water = 7:3, V/V), was introduced to a 20 mg sample, and mixed using vortexing for a duration of 3 min. The sample underwent sonication in an ice bath for a period of 10 min, succeeded by vortexing for 1 min. Subsequently, it was placed in a − 20°C environment for 30 min. Following that, the sample was centrifuged at 12000 rpm for 10 min at 4°C. And the sediment was discarded, the resulting supernatant was centrifuged at 12000 rpm for a duration of 3 min at 4°C. Subsequently, 200 μL aliquots of the supernatant were transferred for analysis using Liquid Chromatograph Mass Spectrometer (LC–MS). All samples were acquired using the LC–MS in strict adherence to the prescribed machine protocols and procedures. The analytical conditions were according to the following parameters, Ultra Performance Liquid Chromatography: column, Waters ACQUITY UPLC HSS T3 C18 (1.8 μm, 2.1*100 mm); column temperature, 40°C; flow rate, 0.4 mL/min; injection volume, 2 μL; solvent system, water (0.1% formic acid): acetonitrile (0.1% formic acid); gradient program, 95:5 V/V at 0 min, 10:90 V/V at 11.0 min, 10:90 V/V at 12.0 min, 95:5 V/V at 12.1 min, 95:5 V/V at 14.0 min.

### 2.5 The use of machine learning to categorize specific states and find possible specific-related biomarkers

The random forest algorithm was applied to train sample classifiers for distinguishing health conditions employing Significantly different microbiomes and metabolites in SAR and the healthy group, respectively. We used the “ranger” R package (v0.12.1) to build the random forest technique using the default hyperparameters except for 5,000 trees in each classification job. A fivefold cross-validation method assessed the RF models’ prediction performance. We further confirmed the performance via the 50–50 training and testing divides. The findings showed the accuracy in the 50% holdout test set and compared the ultimate performance (accuracy) across several cross-validation methods.

We developed disease classifiers utilizing a range of simplified sets of microbiological characteristics and evaluated their performance to ascertain the optimal number of features that might optimize the model performance. The goal is to track the rise or peak in prediction accuracy as more characteristics are incorporated into a classification model.

### 2.6 Statistical analysis and visualization of data

The statistical analyses were conducted utilizing the R (v4.2.2) software. The data are reported as mean ± Standard Error of Mean (SEM). Differential abundances of the genera were assessed utilizing both the Wilcoxon rank-sum test and the Kruskal-Wallis test. Genera with a *p*-value less than 0.05 were deemed significantly different. Principal coordinate analysis (PCoA) was carried out utilizing the “ade4” package to explore the multivariate patterns in the data. Line charts, box plots, volcano plots, and bar charts were generated using the “ggplot2” package for data visualization. Additionally, the construction of the heatmap was accomplished using the “pheatmap” package. The *p*-values underwent filtering and correction through the implementation of the “DESeq2” package within the bubble diagram. The inference of microbial ecological networks was accomplished by employing Spearman’s rank correlation coefficient using the metagenomic sequencing data. Traceability of metabolites using Metorigin software^1^ (Yu et al., 2022). These networks were subsequently visualized in Cytoscape (Version 3.10.0) to facilitate meticulous analysis and interpretation.

## 3 Result

### 3.1 Basic characteristics of clinical features of participants

**Figure 1:**
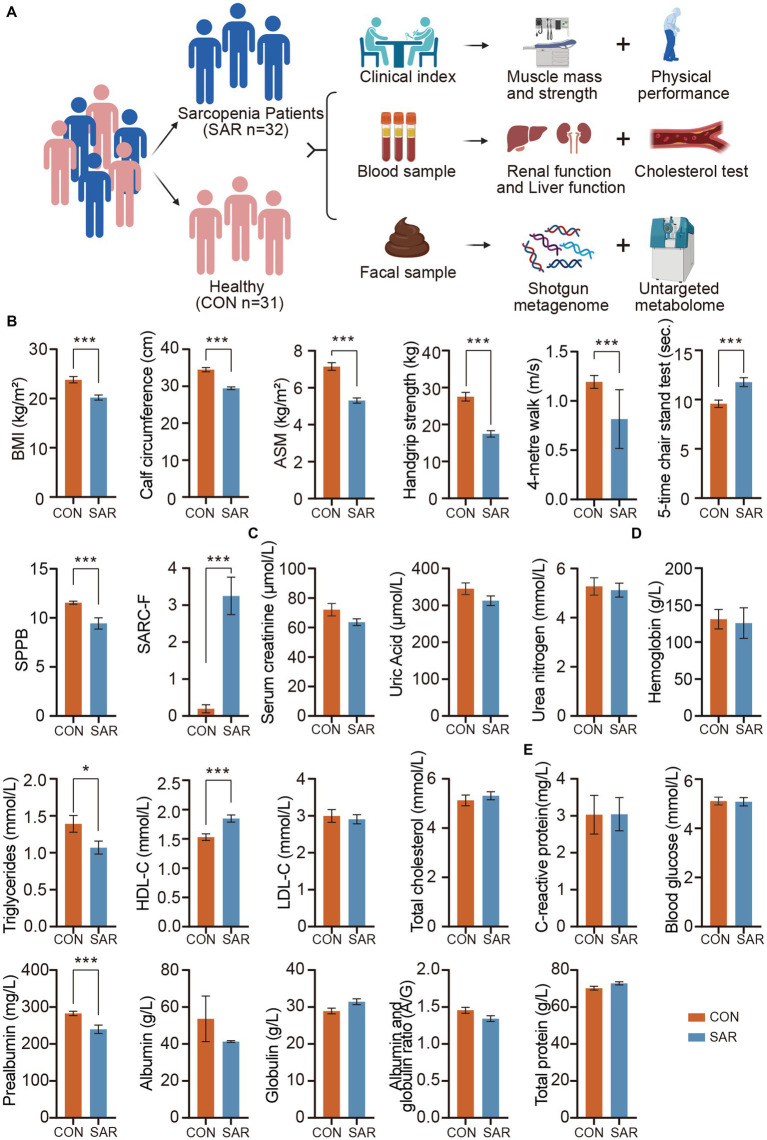
Significantly different in clinical indexes between the SAR and the Health. **(A)** The experimental design. A total of 63 human subjects were divided into two groups according to disease states: healthy control (CON) and sarcopenia group (SAR). Meanwhile, the fecal metagenome and metabolome of the subjects were measured. **(B)** Comparison of Muscle Mass and Physical Performance among the Subjects. **(C)** Kidney function tests based on blood samples. **(D)** Cholesterol tests and HGB based on blood samples. **(E)** Liver function tests based on blood samples. Wilcoxon rank-sum test, ^*^p < 0.05, ^**^p < 0.01, ^***^p < 0.001, error bar: mean ± SEM.

In this study, 63 participants were recruited and separated into two groups; group one, the healthy control group (CON, *n* = 31), and group two, the sarcopenia patient group (SAR, *n* = 32) (Figure 1A). The basic characteristics of these subjects, age, sex, height, and sleep quality, were recorded in Supplementary Table S1. The age distribution of elderly people with sarcopenia was 62–88, and healthy elderly people were 60–79. Meanwhile, we recorded the subjects’ basic motor abilities according to the Strength, assistance with walking, rising from a chair, climbing stairs, and falls (SARC-F) questionnaire.

Obviously, in individuals with sarcopenia, there was a notable reduction in both body mass index (BMI) and calf circumference compared to the healthy group (Figure 1B). From a more detailed perspective, participants with sarcopenia exhibited significant decreases in appendicular skeletal muscle mass (ASM), handgrip strength, 4-metre walking speed, short physical performance battery (SPPB), and SARC-F scores, while the 5-time chair stand test showed a significant increase compared to the control group. The changes in these indexes reveal a comprehensive profile of muscle mass, muscle strength, and physical performance in the poor physical quality of patients with sarcopenia. These underlying physical differences directly map to changes in clinical serum indexes. First, we checked the kidney-related indexes in patients with sarcopenia. There were no significant differences in Serum creatinine (SCR), Uric Acid (UA), and Blood Urea nitrogen (BUN) between CON and SAR groups (Figure 1C). C-reactive protein (CRP), hemoglobin (HGB), total protein (TP) and blood glucose (GLU) in SAR were also within healthy limits (Figures 1D,E). In contrast, prealbumin (PA) was significantly lower in SAR (Figure 1E). In addition, we found a slight increase in serum globulin (GLB) and a slight decrease in serum Albumin (ALB) and albumin to globulin ratio (A/G) in patients with sarcopenia, although the absence of the significant difference between SAR and CON.

For lipid metabolism, we noted a significant decrease in triglycerides (TG) and a significant increase in high-density lipoprotein cholesterol (HDL-C) in patients with sarcopenia (Figure 1D). Similar results have been reported in other clinical studies of sarcopenia (Wang et al., 2020; Jiang et al., 2023).

### 3.2 Sarcopenia alters the gut microbiota

**Figure 2:**
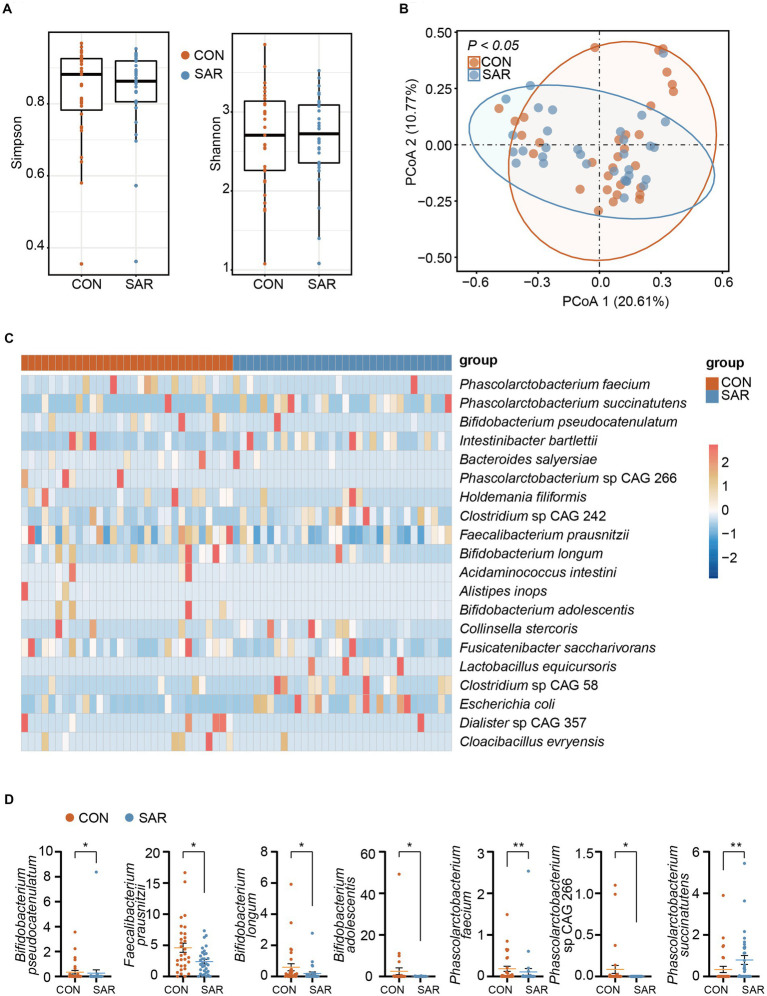
The metagenome profile of intestinal microbial between Sarcopenia and Healthy. **(A)** Alpha diversity (Simpson and Shannon index) of the intestinal microbial colony. **(B)** Principal coordinate analysis (PCoA) showed the microbial composition changes between SAR and Healthy based on bray-Curtis distance. The different colored dots indicate the individuals’ intestinal microbial structure in each group. **(C)** All species with significant differences (*p* < 0.05) in intestinal microbial between the SAR and the CON by applying the Wilcoxon rank-sum test. More blue means less abundant; more red means more abundant. **(D)** Comparison of the abundance of some beneficial or harmful species between the two groups. Wilcoxon rank-sum test, ^*^p < 0.05, ^**^p < 0.01, ^***^p < 0.001, error bar: mean ± SEM.

We next sought to explore the difference in the intestinal microbiome of patients between CON and SAR. The alpha diversity of the gut microbiome was quantified based on profiles of metagenomics species-level (Figure 2A). There was no significant difference in the Shannon index and Simpson index between the CON and SAR groups. This indicates that there is no significant difference in species richness of the gut microbiota between sarcopenic and healthy populations. However, from the perspective of β-diversity of the microbiome, principal coordinates analysis (PCoA) based on Bray-Curtis distances demonstrated that sample points representing the two groups showed significantly different separation trends (*p* < 0.05) (Figure 2B). This suggests that the microbiome structure is significantly different between patients with sarcopenia and healthy older adults.

Since we have already observed that the gut microbiome structure was disordered in SAR, we further screened for differences in species between the two groups with the aim of quantifying in more detail the differences in the microbiome between the two groups. The proportional abundance of *Bifidobacterium longum*, *Bifidobacterium pseudocatenulatum*, and *Bifidobacterium adolescentis* was significantly decreased in the SAR group (Figure 2C). In addition, several potentially beneficial species were found to be significantly declining in abundance such as *Faecalibacterium prausnitzii*, *Fusicatenibacter saccharivorans*, and *Phascolarctobacterium faecium*. In contrast, the relative abundance of some non-beneficial species, such as *Clostridium* sp. CAG 242, *Phascolarctobacterium succinatutens*, and *Clostridium* sp. CAG 58, showed a significantly higher abundance in SAR compared to the CON group.

### 3.3 Functional differences in the gut microbiome of the sarcopenia population

**Figure 3:**
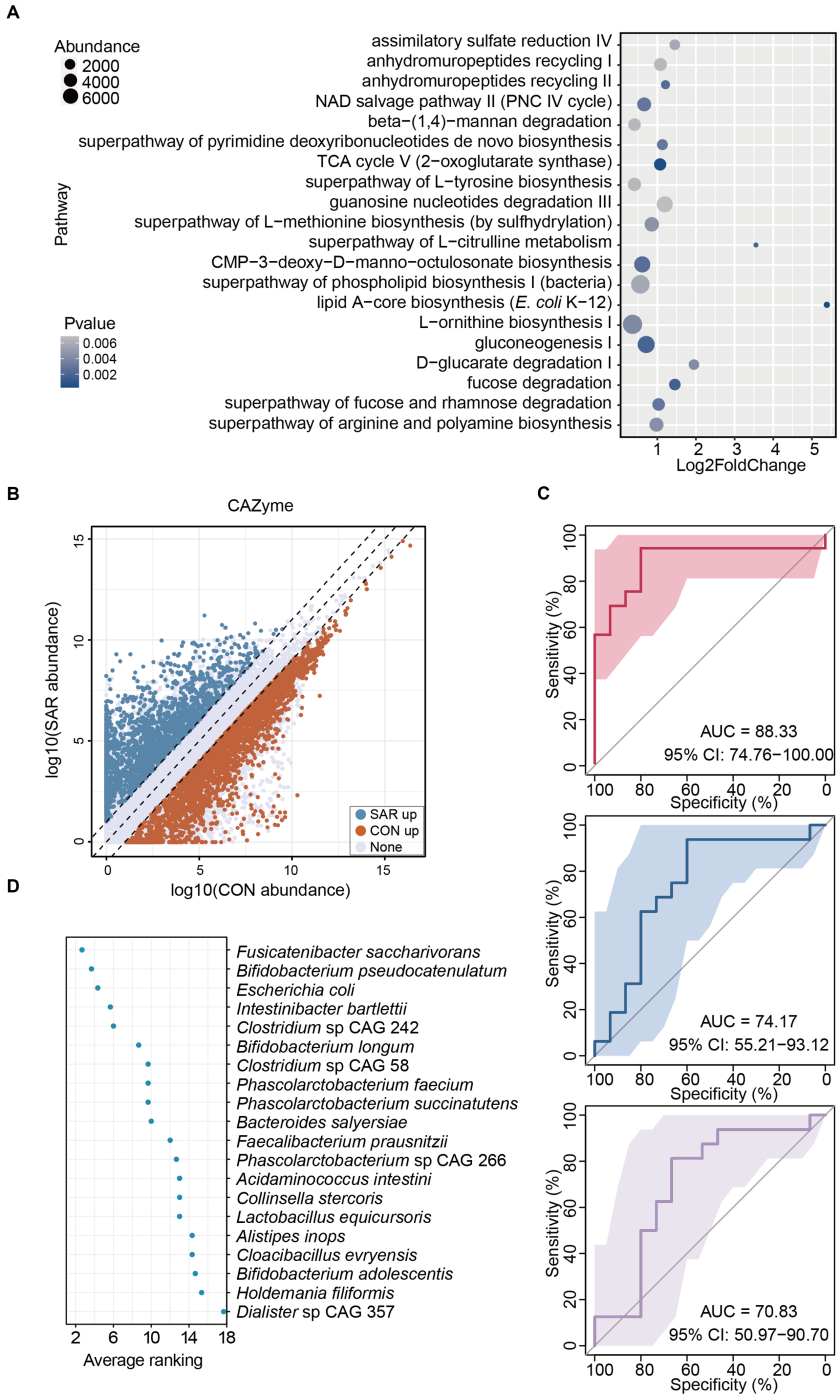
Alterations in gut microbiome function in patients with sarcopenia. **(A)** The significantly different pathways between the SAR and the CON. Based on the results from the shotgun metagenomic sequencing, metabolic pathway analysis was executed. The top 20 pathways with significant differences (*p* < 0.05) were selected. The enrichment abundance in the SAR group is higher than that in the Health group, according to the log2foldchange value, which must be larger than 0. **(B)** Significant variations in CAZyme were annotated between the two groups. **(C)** Classification of the SAR group and the health group using bacterial species-level biomarkers based on shotgun metagenomic sequencing data in the three consecutive random samplings. The area under the curve (AUC) and the receiver operating characteristic (ROC) curve were calculated in the training and test groups. **(D)** Ranking the importance of each species to the prediction model; the smaller the ranking, the greater the importance.

After clarifying the structural and species-level differences in the gut microbiome of sarcopenia, we have further analyzed the functionality of the gut microbiome in both cohorts. The top 20 metabolic pathways with significant differences were screened to gain more insight into the possible functional impact of different microbial structures (Figure 3A). Surprisingly, all the top 20 metabolic pathways were enriched in the SAR group, such as ornithine, methionine, and tyrosine arginine and polyamine biosynthesis, which are involved in the metabolic function of amino acids. In addition, fucose degradation, fucose and rhamnose degradation, β-(1,4)-mannan degradation, and TCA cycle V were also significantly upregulated in SAR compared with CON. However, an increase in the functional pathway of partial sugar degradation does not fully represent a concomitant increase in the utilization of carbohydrates. Therefore, we proceeded to compare the abundance of functional genes for carbohydrate-active enzymes in SAR and CON metagenome samples based on the Carbohydrate-Active enzymes (CAZyme) database. The number of significantly up-regulated CAZyme genes in the CON group (3159) was well above that in the SAR group (1891) (Figure 3B). This implied that patients with sarcopenia have a significantly poorer ability in the intestine to synthesize or break down complex carbohydrates and sugar complexes than healthy people.

These results indicate that the gut microbiome of patients with sarcopenia is structurally and functionally distinct from that of the healthy population. Thus, the microbiota in the gut can screen patients with sarcopenia to some extent. Therefore, we chose the 20 species that were screened with significant differences, as noted above. A supervised classification model to screen for sarcopenia was trained based on a random forest approach. The accuracy performance of the model was further validated by 50–50% training and testing of the segmentation. In three consecutive random sample validations, the classifier could distinguish to some extent between healthy people and patients with sarcopenia at the level of intestinal species, although there was some fluctuation in accuracy from low to high, the area under curve (AUC) = 70.83–88.33% (Figure 3C). Also, we ranked 20 differential species based on their contribution to this classifier based on three random samples (Figure 3D). The top 3 highest average rankings were *Fusicatenibacter saccharivorans*, *Bifidobacterium pseudocatenulatum*, and *Escherichia coli*, while the lowest were *Bifidobacterium adolescentis*, *Holdemania filiformis* and *Dialister* sp. CAG 357.

### 3.4 Sarcopenia patients have a particular fecal metabolite profile.

**Figure 4:**
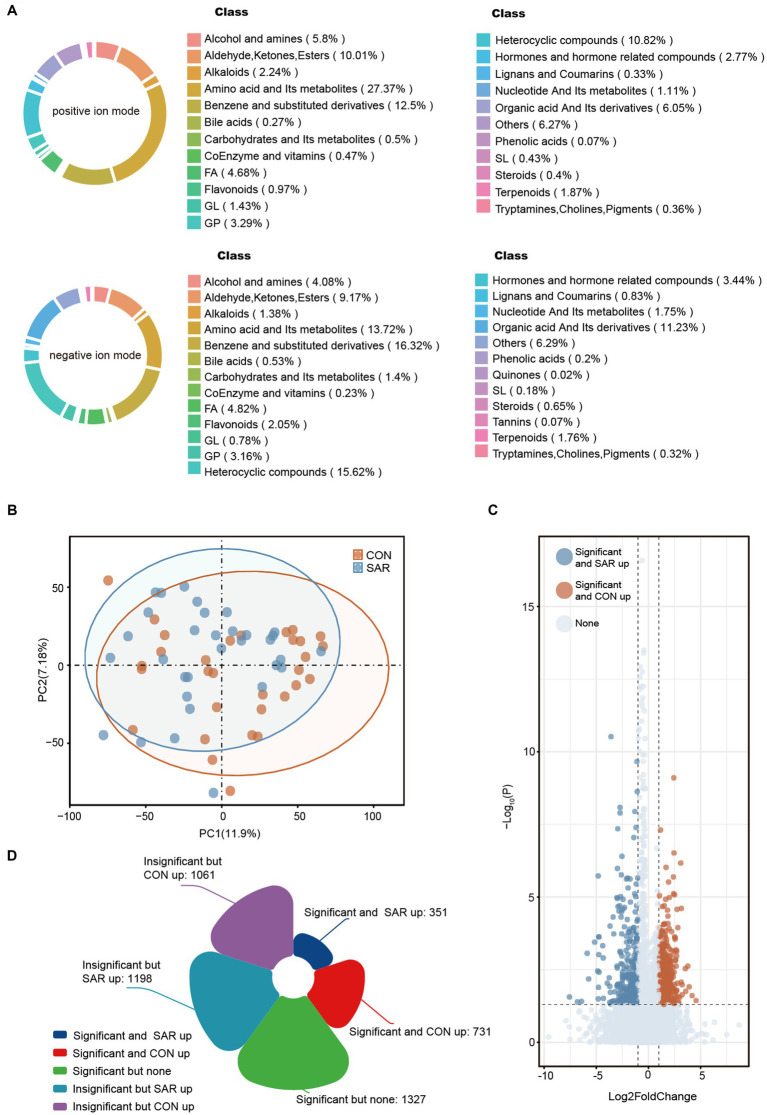
Metabolic profile of intestinal microbial between SAR and CON. **(A)** Based on the anionic and cationic models, secondary metabolite classification was performed for all fecal metabolites annotated at the metabolome level. **(B)** Principal Component Analysis (PCA) showed the metabolic composition changes between SAR and Healthy based on the bray-Curtis distance. The different colored dots indicated the individuals’ intestinal structure of metabolites in each group. **(C)** Differential metabolites between SAR group and CON group. The closer the point was to the left/right, the more significant the difference in abundance multiples between the two groups. The closer the point was to the top, the smaller the *p*-value. The blue/yellow dots on the left and right sides indicate metabolites that were significantly upregulated in the SAR group/significantly upregulated in the CON group, respectively. **(D)** Number of significantly altered fecal metabolites in patients with sarcopenia compared to controls.

**Figure 5:**
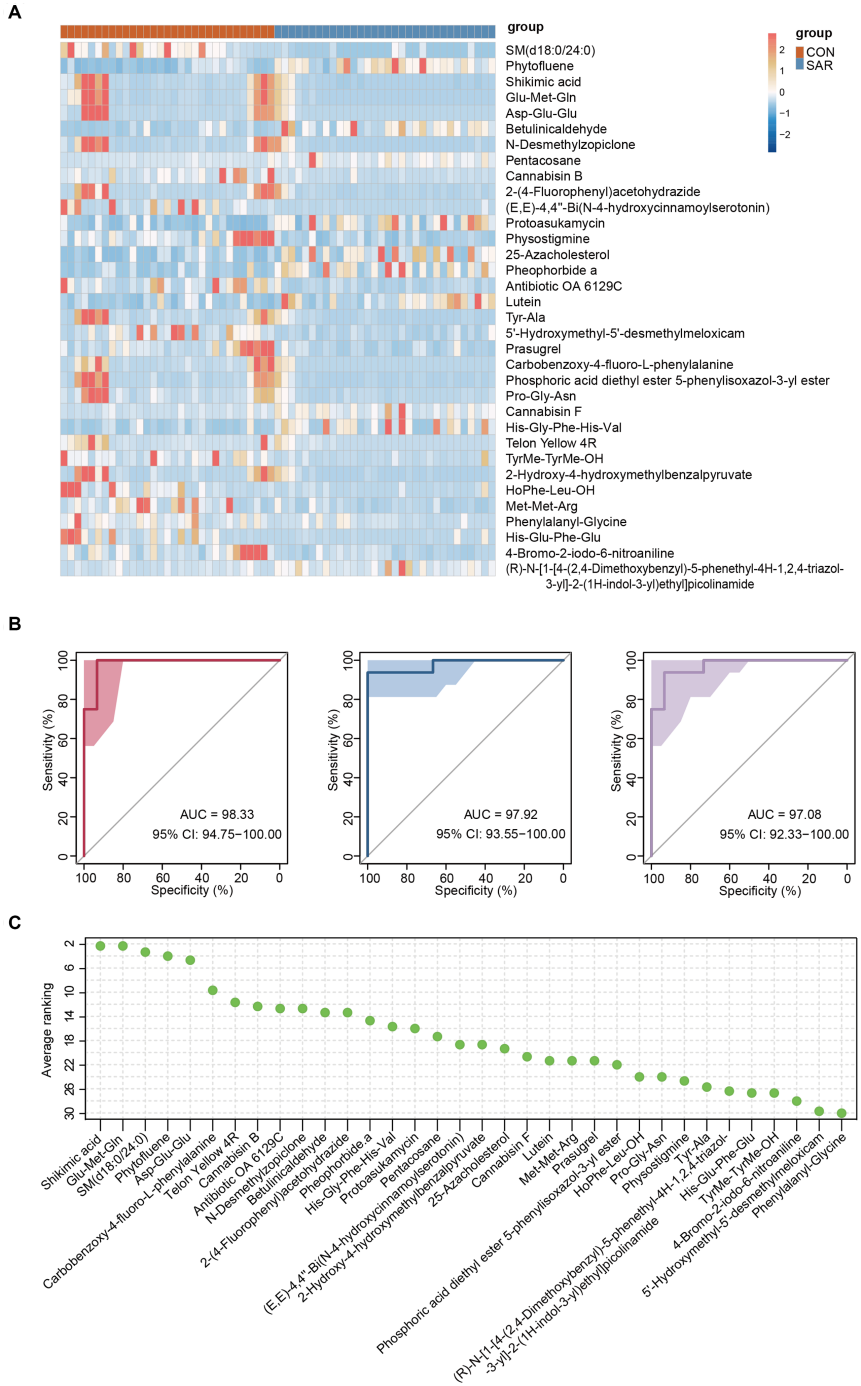
Differential fecal metabolites and metabolomic model predictions. **(A)** The relative abundance content of 34 metabolites of biomarkers. Darker blue means lower abundance, and darker red means higher abundance. **(B)** Classification of the SAR group and the CON group using 34 metabolites as biomarkers in the three consecutive random samplings. AUC and ROC are both in the training and test groups. **(C)** Ranking the importance of each metabolite to the prediction model; the smaller the ranking, the greater the importance.

A total of 13,025 metabolites in fecal samples from two groups of 63 subjects was identified based on positive/negative ion mode. The ratio of metabolite classes differs in the two ionic modes, mainly amino acid and its metabolites (13.72–27.37%), benzene and substituted derivatives (12.5–16.32%) and heterocyclic compounds (10.82–15.62%) (Figure 4A). From the structure of the metabolome, the samples representing the SAR and CON groups did not form a distinctly separated trend based on principal component analysis (PCA) (Figure 4B). Compared to the gut microbiome, the structural differences in the intestinal metabolome were not significant. However, in view of specific metabolite levels, there were still many differential metabolites enriched in the CON and SAR groups, respectively (Figure 4C). There were 731 differential metabolites significantly up-regulated in the CON group (means significantly down-regulated in the SAR group) and 351 differential metabolites significantly up-regulated in the SAR group (Figure 4E). To locate the more critical metabolites, we performed further screening with |Log2FoldChange| > 2 and P-adjust>0.01. Finally, 34 highly significant differential metabolites were screened out (Figure 5A). Some potentially beneficial substances, such as Shikimic acid and SM (d18:0/24:0), were significantly down-regulated in the SAR group. Interestingly, short peptides such as Tyr-Ala, Pro-Gly-Asn, Met-Met-Arg, and His-Glu-Phe-Glu were significantly decreased in the SAR group, which may indicate better utilization of these proteins in healthy populations compared to patients with sarcopenia. Although the separation in the structure of the metabolome was not significant, we still found differences in specific metabolite species between the two groups.

Therefore, the same approach was used to build classification models aimed at these differential metabolites. Excitingly, the accuracy of the classification models established for the differential metabolites was consistently above 97% (AUC = 97.08–98.33%) in three consecutive random sample validations (Figure 5B). And among these metabolites, highest three contributing metabolites were Shikimic acid, Glu-Met-Gln and SM (d18:0/24:0), while lowest three were 4-Bromo-2-iodo-6-nitroaniline, 5’-Hydroxymethyl-5′-desmethylmeloxicam and Phenylalanyl-Glycine (Figure 5C).

### 3.5 Mechanisms of gut microbiome affecting sarcopenia through metabolites in sarcopenia

**Figure 6:**
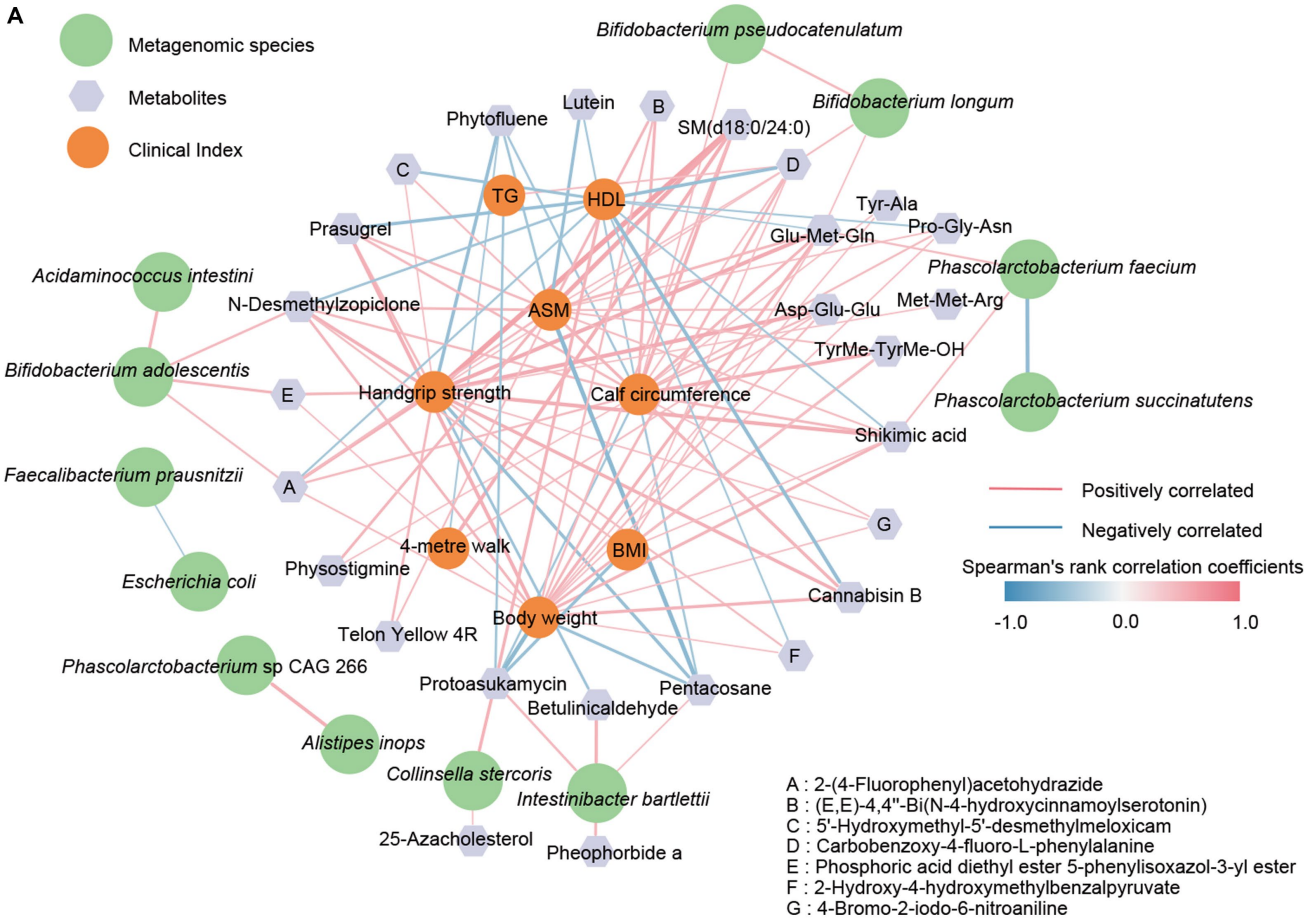
The correlation network between clinical indicators, metagenomic species, and signature metabolites. **(A)** The R-value greater than 0.4 or less than −0.4 was selected based on Spearman’s rank correlation coefficient. The line width (the thicker the line, the stronger the correlation) and color (red, positive; blue, negative) were proportionate to the strength of the correlation.

As mentioned above, patients with sarcopenia and healthy older adults showed disturbances in the gut microbiome and fecal metabolome, further performing significant differences in some clinical indexes. These results prompted us to explore specific associations between the different omics further. Therefore, a network was generated by the Spearman rank correlation coefficient to show the association between bacterial species, metabolites, and clinical indexes associated with sarcopenia (Figure 6). As we emphasized previously, *Bifidobacterium longum* and *Bifidobacterium pseudocatenulatum* were significantly decreased in the intestine of the sarcopenic group, and there may be this mutually reinforcing effect relationship between them. The latter, *Bifidobacterium pseudocatenulatum*, was then linked to calf circumference and showed a positive correlation. We also observed that *Bifidobacterium longum* showed a positive correlation with Glu-Met-Gln and Carbobenzoxy-4-fluoro-L-phenylalanine, while both may promote the improvement of TG, AMS, Handgrip strength, BMI, and calf circumference. In contrast, Protoasukamycin, positively associated with *Collinsella stercoris*, may inhibit TG elevation. In addition, *Phascolarctobacterium faecium* was found to possibly restrain *Phascolarctobacterium succinatutens* and promote the production of Shikimic acid. Based on the metabolite traceability by Metorigin, we further clarified that *Phascolarctobacterium faecium* was able to produce Shikimic acid. Then, elevated shikimic acid was likewise linked to AMS, Handgrip strength, BMI, and calf circumference (positive correlation). Thus, the down-regulation of certain crucial species in the gut of patients with sarcopenia may affect their Clinical indexes related to muscle mass and physical performance through the combined action of multiple metabolites.

## 4 Discussion

In this study, we investigated the relationship between the gut microbiome and metabolome in sarcopenia patients. The gut microbiome and metabolome of SAR patients have obviously altered in comparison to healthy CON, according to a combined analysis of shotgun metagenome and untargeted metabolomics data from fecal samples and clinical parameters of the participants. We used shotgun metagenomic sequencing and untargeted metabolomics to identify 20 microbial species (including *Bifidobacterium pseudocatenulatum*, *Bifidobacterium longum*, *Bifidobacterium adolescentis*, *Phascolarctobacterium succinatutens*, *Phascolarctobacterium faecium* and *Faecalibacterium prausnitzii*) and 34 metabolites (including organic acid and small peptide), and that were used as the biomarker to distinguish patients from healthy subjects.

Only a few researchers have yet, investigated the relationship between human sarcopenia (not based on animal models, which are difficulties and restrictions entailed in extrapolating complicated human illnesses from reductionist animal models) and the gut microbiome. Several bacterial genera were shown to be different between sarcopenia patients and controls in several studies (Ticinesi et al., 2017; Picca et al., 2018); 16S rRNA sequencing was reliable but difficult to distinguish between closely related species. Only a small number of studies discovered statistically significant differences in the gut microbiota composition by employing shotgun metagenomic sequencing between sarcopenic patients and healthy adults (Ticinesi et al., 2020; Wang et al., 2022), and only the prediction of metagenomic function was used, but no metabolomics analysis was performed.

Our study found that *Bifidobacterium pseudocatenulatum*, *Bifidobacterium longum*, and *Bifidobacterium adolescentis* were decreased in participants of sarcopenia. *Bifidobacterium*, as probiotics, has been illustrated might be effective for aging due to its ability to modulate oxidative stress and inflammation (Chen et al., 2021; Fang et al., 2021). In addition, Shujie C. et al. reported that supplementation with *Bifidobacterium adolescentis* resulted in an enhancement in skeletal muscle catalase enzyme activity and suppressed cellular senescence in mouse embryonic fibroblasts, and aroused alterations in oxidative stress-associated metabolites (Chen S. et al., 2020). Disturbance of gut microbiota was detrimental to the health of the host (Zhang et al., 2015). Furthermore, intestinal microbiota issues are frequently characterized by the considerable reduction of *Faecalibacterium prausnitzii* and *Bifidobacterium* (Vijay and Valdes, 2022; Chen et al., 2023); these reports are in according with our finding of decreased *Bifidobacterium pseudocatenulatum*, *Bifidobacterium longum*, *Bifidobacterium adolescentis*, and *Faecalibacterium prausnitzii* in the SAR group. We found that the abundance of *Phascolarctobacterium faecium* was down-regulated in the SAR group as a potential probiotic that may be associated with intestinal detoxification. On the contrary, *Phascolarctobacterium succinatutens*, which was correlated with the risk of Multiple sclerosis (MS) and metabolic dysfunction-associated fatty liver disease (MAFLD) and positively correlated with disability severity in MS patients (Cantoni et al., 2022), was enriched in SAR. *Faecalibacterium Prausnitzii*, *Bifidobacterium adolescentis*, *Bifidobacterium longum*, and *Bifidobacterium pseudocatenulatum* are producers of short-chain fatty acids (SCFAs) (Heinken et al., 2014; Turroni et al., 2016). SCFAs have been reported to have a variety of health-promoting effects, such as regulating bone mass (Lucas et al., 2018), regulating immunity (Yang et al., 2020), and even improving sarcopenia (Lv et al., 2021; Han et al., 2022). Therefore, a decrease in the abundance of these keystone species may be accompanied by a decrease in the abundance of SCFAs (Chen et al., 2022; Han et al., 2022). Spearman correlation analysis showed that the abundance of *Phascolarctobacterium faecium* and *Phascolarctobacterium succinatutens* were correlated negatively. These findings imply that the intestinal bacteria imbalance may be better by supplementing with similar or related probiotics. This also reflects the necessity of identifying bacteria down to the species level.

Our results from the fecal metabolome showed some short peptides such as Tyr-Ala, Pro-Gly-Asn, Met-Met-Arg, and His-Glu-Phe-Glu in SAR were significantly lower than CON, suggesting that the intestinal amino acid metabolism of sarcopenia patients is abnormal and sarcopenia patients may have malabsorption or malnutrition. Tyrosine (Tyr) and phenylalanine (Phe) have been shown in earlier research to promote the anabolism of muscle proteins and increase muscular performance (Volpi et al., 2003). Additionally, tryptophan (Trp), an essential amino acid, has the potential to regulating skeletal muscle mass (Ryan et al., 2008). Shikimic acid, a significant intermediary in the biosynthesis of aromatic amino acids in bacteria, is a precursor to the aromatic rings of Phe, Tyr, and Trp (Kumar and Goel, 2019). We found that the reduction of shikimic acid in the SAR group was significant, indicating abnormal Shikimic acid metabolism in this group. Moreover, this anomaly may affect the synthesis of these three amino acids, resulting in a reduction in synthesis, which can affect muscle mass and function. Spearman’s correlation analysis showed a significant positive correlation between *Phascolarctobacterium faecium* and shikimic acid, while the metabolite traceability-based approach directly confirmed that *Phascolarctobacterium faecium* was able to produce shikimic acid. Based on the above available evidence and the correlation between metabolites, bacteria, and the indexes, we found that *Phascolarctobacterium faecium* could promote shikimic acid of fecal, which affects Phenylalanine, tyrosine, and tryptophan biosynthesis, and thus clinical indexes. In addition, *Bifidobacterium longum* may affect the intestinal metabolism of certain small peptides (such as Glu-Met-Gln) and, thus, the clinical indicators of sarcopenia.

**Figure 7:**
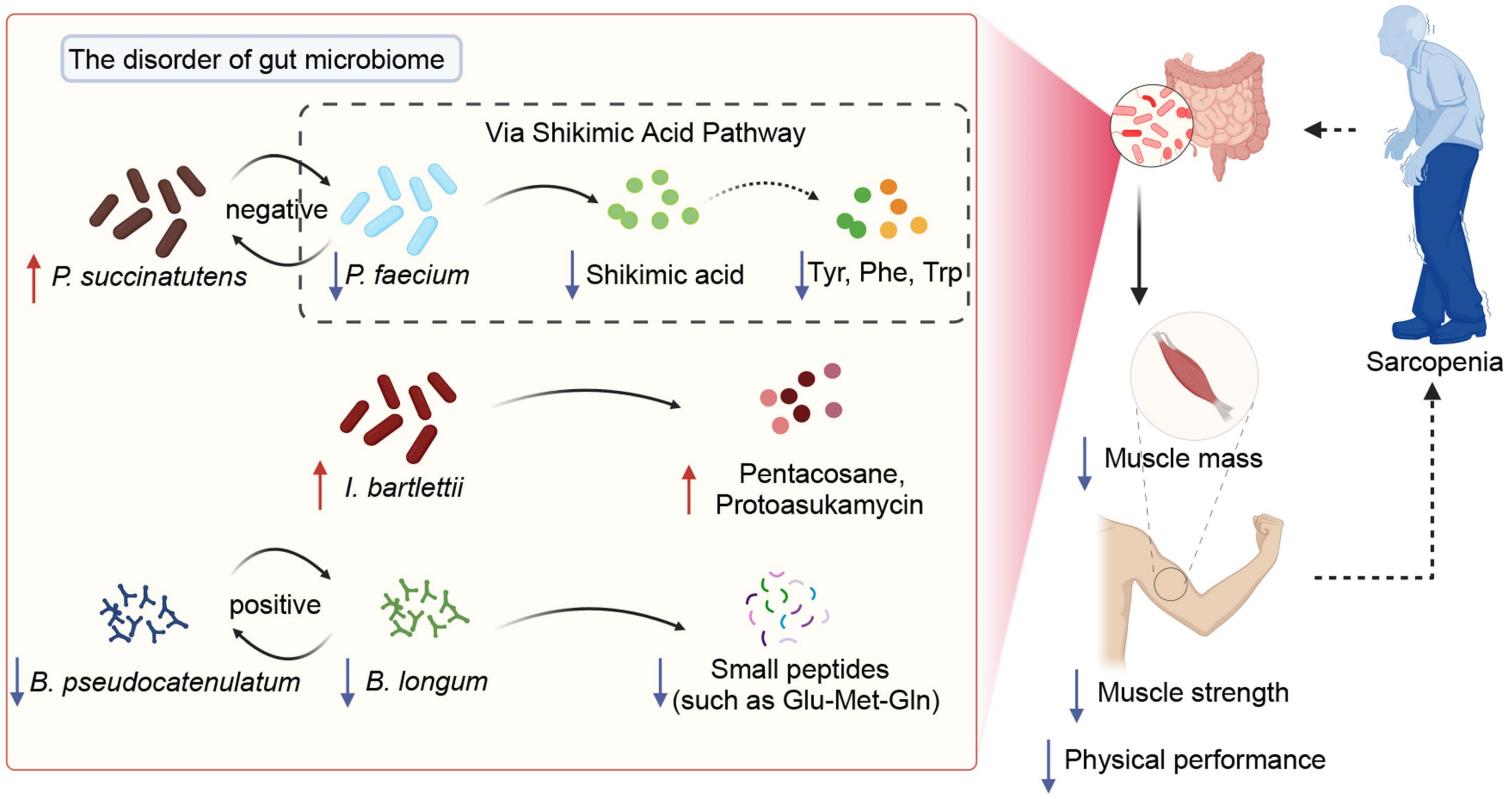
The potential mechanisms of gut microbiota modulate sarcopenia through specific metabolites.

In this study, the characterization and function of the gut microbiome of patients with sarcopenia were depicted based on gut metagenome and metabolomics. This study showed that patients with sarcopenia had a disturbed gut microbiome with a significant decrease in beneficial species such as *Bifidobacterium pseudocatenulatum*, *Bifidobacterium longum*, and *Bifidobacterium adolescentis*. This phenomenon directly leads to a decrease in functions such as carbohydrate utilization by the intestinal microbiota, as well as a decline in potentially beneficial substances (such as shikimic acid and small peptides) and a rise in harmful substances (such as pentacosane and protoasukamycin). Supervised classification models were drawn to differential biomarkers at the intestinal species level and metabolite level, respectively. Importantly, we propose a potential mechanism that *Phascolarctobacterium faecium* affects ASM, calf circumference, handgrip strength, and BMI via Shikimic acid metabolites (Figure 7). This study expands the understanding of the gut microbiome and metabolome profiles in sarcopenia and provides a strong base and reference for the targeted treatment of sarcopenia.

## Data availability statement

The datasets presented in this study can be found in online repositories. The names of the repository/repositories and accession number(s) can be found at: NCBI - PRJNA992232, MetaboLights (MTBLS8149).

## Ethics statement

The studies involving humans were approved by The Ethics Committee of the Hainan General Hospital (2021-317). The studies were conducted in accordance with the local legislation and institutional requirements. The participants provided their written informed consent to participate in this study. Written informed consent was obtained from the individual(s) for the publication of any potentially identifiable images or data included in this article.

## Author contributions

YH: Conceptualization, Writing – review & editing. WC: Data curation, Visualization, Writing – original draft. TF: Methodology, Writing – review & editing. ZZ: Supervision, Visualization, Writing – review & editing. MZ: Supervision, Writing – review & editing.

## Footnotes

^1^ http://metorigin.met-bioinformatics.cn/
