# Supplementary material for: Metabolites of the gut microbiota may serve as precise diagnostic markers for sarcopenia in the elderly
Source: Front Microbiol. 2023 Dec 21;14:1301805. doi: 10.3389/fmicb.2023.1301805 (PMC10768011; doi:10.3389/fmicb.2023.1301805)
Supplement: Supplementary file 1 [file Table_1.docx]

**Table S1 Information for participants.**

| Subject ID | Gender | Age | Height (cm) | Weight (kg) |
| --- | --- | --- | --- | --- |
| CON1 | Female | 73 | 148 | 39.8 |
| CON2 | Male | 75 | 163 | 63.3 |
| CON3 | Female | 66 | 158 | 58.2 |
| CON4 | Female | 65 | 150 | 41.1 |
| CON5 | Male | 69 | 155 | 60.6 |
| CON6 | Male | 74 | 166 | 71.15 |
| CON7 | Female | 66 | 150 | 62.35 |
| CON8 | Male | 73 | 165 | 80.6 |
| CON9 | Female | 63 | 157 | 51.9 |
| CON10 | Male | 72 | 159 | 53.25 |
| CON11 | Male | 78 | 175 | 68.05 |
| CON12 | Female | 72 | 153 | 46 |
| CON13 | Female | 73 | 156 | 52.05 |
| CON14 | Female | 66 | 157 | 53.5 |
| CON15 | Male | 67 | 172 | 65.4 |
| CON16 | Female | 68 | 162 | 48.5 |
| CON17 | Female | 64 | 157 | 64.8 |
| CON18 | Female | 70 | 153.5 | 48.4 |
| CON19 | Female | 68 | 150 | 58.85 |
| CON20 | Female | 69 | 153 | 56.7 |
| CON21 | Female | 77 | 152 | 50.7 |
| CON22 | Male | 60 | 160 | 57 |
| CON23 | Male | 75 | 170 | 75.4 |
| CON24 | Female | 68 | 150 | 68.8 |
| CON25 | Male | 67 | 172 | 62.8 |
| CON26 | Male | 68 | 162 | 81.7 |
| CON27 | Female | 71 | 163 | 73.5 |
| CON28 | Female | 72 | 166 | 64.9 |
| CON29 | Female | 79 | 150 | 48.9 |
| CON30 | Male | 72 | 163 | 73.2 |
| CON31 | Female | 64 | 158 | 68.6 |
| SAR1 | Female | 77 | 145 | 43.85 |
| SAR2 | Male | 68 | 158 | 54.2 |
| SAR3 | Male | 78 | 147 | 49.7 |
| SAR4 | Male | 70 | 151 | 47.65 |
| SAR5 | Female | 73 | 144 | 47.2 |
| SAR6 | Female | 69 | 150 | 32.75 |
| SAR7 | Female | 78 | 142.5 | 42.95 |
| SAR8 | Female | 81 | 148 | 45.75 |
| SAR9 | Female | 77 | 150 | 41.15 |
| SAR10 | Female | 76 | 162 | 47.6 |
| SAR11 | Female | 74 | 147 | 51.8 |
| SAR12 | Female | 79 | 147 | 37.9 |
| SAR13 | Female | 82 | 150 | 45.55 |
| SAR14 | Female | 62 | 140 | 40.25 |
| SAR15 | Female | 70 | 152.5 | 43.45 |
| SAR16 | Male | 71 | 165 | 37.8 |
| SAR17 | Female | 66 | 152 | 38.95 |
| SAR18 | Male | 78 | 147 | 39.2 |
| SAR19 | Male | 75 | 157 | 51.1 |
| SAR20 | Female | 77 | 143 | 54 |
| SAR21 | Female | 71 | 148 | 39.5 |
| SAR22 | Female | 84 | 145 | 50.6 |
| SAR23 | Female | 78 | 150 | 44.15 |
| SAR24 | Female | 70 | 141 | 32.85 |
| SAR25 | Female | 73 | 150 | 39.5 |
| SAR26 | Female | 81 | 155 | 45.6 |
| SAR27 | Male | 73 | 157 | 56.1 |
| SAR28 | Female | 69 | 155 | 42.85 |
| SAR29 | Female | 83 | 140.5 | 47.55 |
| SAR30 | Female | 81 | 141 | 45.55 |
| SAR31 | Female | 88 | 150 | 45.75 |
| SAR32 | Female | 72 | 149 | 54 |
